# Jejunal adenocarcinoma with isolated mesorectal metastasis: a case report of a rare entity

**DOI:** 10.1093/jscr/rjag226

**Published:** 2026-04-04

**Authors:** Filipa Corte-Real, Sara Catarino, Milene Sá, Fernando Valério, Jorge Pereira

**Affiliations:** Serviço de Cirurgia Geral da Unidade Local de Saúde Viseu Dão-Lafões, Av. Rei Dom Duarte, Viseu, 3504-509, Portugal; Clinical Academic Center of Beiras (CACB), Edifício UBImedical, Estrada Municipal 506, Covilhã, 6200-284, Portugal; Serviço de Cirurgia Geral da Unidade Local de Saúde Viseu Dão-Lafões, Av. Rei Dom Duarte, Viseu, 3504-509, Portugal; Clinical Academic Center of Beiras (CACB), Edifício UBImedical, Estrada Municipal 506, Covilhã, 6200-284, Portugal; Serviço de Cirurgia Geral da Unidade Local de Saúde Viseu Dão-Lafões, Av. Rei Dom Duarte, Viseu, 3504-509, Portugal; Clinical Academic Center of Beiras (CACB), Edifício UBImedical, Estrada Municipal 506, Covilhã, 6200-284, Portugal; Serviço de Cirurgia Geral da Unidade Local de Saúde Viseu Dão-Lafões, Av. Rei Dom Duarte, Viseu, 3504-509, Portugal; Clinical Academic Center of Beiras (CACB), Edifício UBImedical, Estrada Municipal 506, Covilhã, 6200-284, Portugal; Serviço de Cirurgia Geral da Unidade Local de Saúde Viseu Dão-Lafões, Av. Rei Dom Duarte, Viseu, 3504-509, Portugal; Clinical Academic Center of Beiras (CACB), Edifício UBImedical, Estrada Municipal 506, Covilhã, 6200-284, Portugal

**Keywords:** small bowel neoplasms, jejunum adenocarcinoma, obscure gastrointestinal bleeding, enterectomy, mesorectum metastasis

## Abstract

Small bowel (SB) neoplasms are rare and often diagnosed at advanced stages, commonly presenting with obstruction or obscure gastrointestinal bleeding (OGB). An 83-year-old man presented with anemia and hematochezia. Imaging revealed an 8 cm SB mass and a 5 cm rectal lesion initially suggestive of GIST. Biopsy confirmed adenocarcinoma. Due to recurrent bleeding, the patient underwent segmental enterectomy and Hartmann’s procedure. Histology showed jejunal mucinous adenocarcinoma with mesorectal metastasis (pT4N0M1). Despite adjuvant chemotherapy and hemostatic radiotherapy for pelvic recurrence, the patient died 7 months postoperatively. SB tumors should be considered in OGB with anemia. Adenocarcinoma is the most common type. Surgery was chosen due to refractory anemia and resectable oligometastatic disease in the mesorectum. Jejunal adenocarcinoma is difficult to diagnose. Surgery remains the main treatment and selected resection of distant metastases may improve survival. Mesorectal involvement is rare and presents additional management challenges.

## Introduction

Small bowel (SB) tumors are rare, accounting for less than 3% of all GI neoplasms and most are malignant [1–3].

Generally, these tumors are diagnosed in the context of obstruction or obscure gastrointestinal bleeding (OGB) or in cases of SB obstruction. OGB, defined as bleeding of unknown origin following endoscopic evaluation, originates in the SB in 75% of cases [4, 5]. Nonspecific symptoms frequently delay diagnosis, resulting in poor prognosis [6–8]. Surgery remains the best therapeutic option [9, 10]. The treatment of metastatic disease is usually systemic. Selected patients may benefit from metastasis resection [8, 11].

## Case report

An 83-year-old Caucasian male was admitted to the ED with hematochezia, altered bowel habits, weight loss, asthenia, and anorexia, without abdominal pain. The patient had a history of hypertension, dyslipidemia and transurethral resection for prostatic pathology. The patient was normotensive. On abdominal examination, a firm, non-tender, palpable mass was identified in the left quadrants. Blood work revealed anemia (hemoglobin 6.4 g/dL). The patient received two units of packed red blood cells.

A upper endoscopy was normal. A subsequent total colonoscopy with terminal ileoscopy revealed a subepithelial lesion 10 cm from the anal verge and traces of blood throughout the entire colon without other lesions.

**Figure 1:**
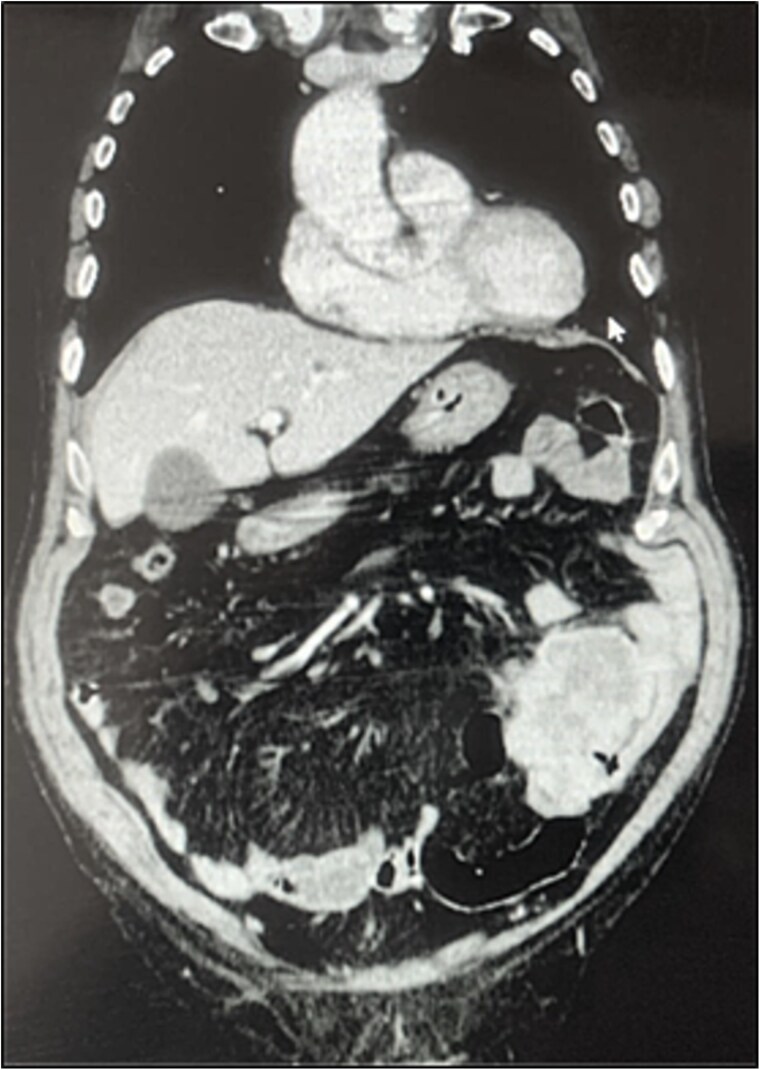
SB mass (coronal plane).

**Figure 2:**
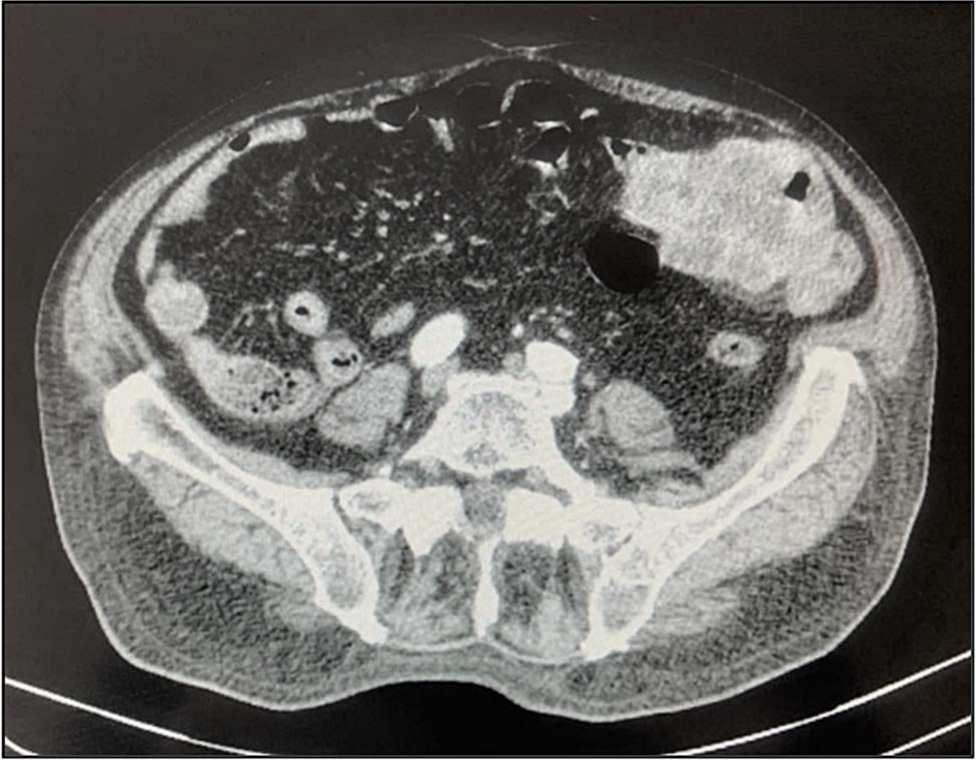
SB mass (axial plane).

**Figure 3:**
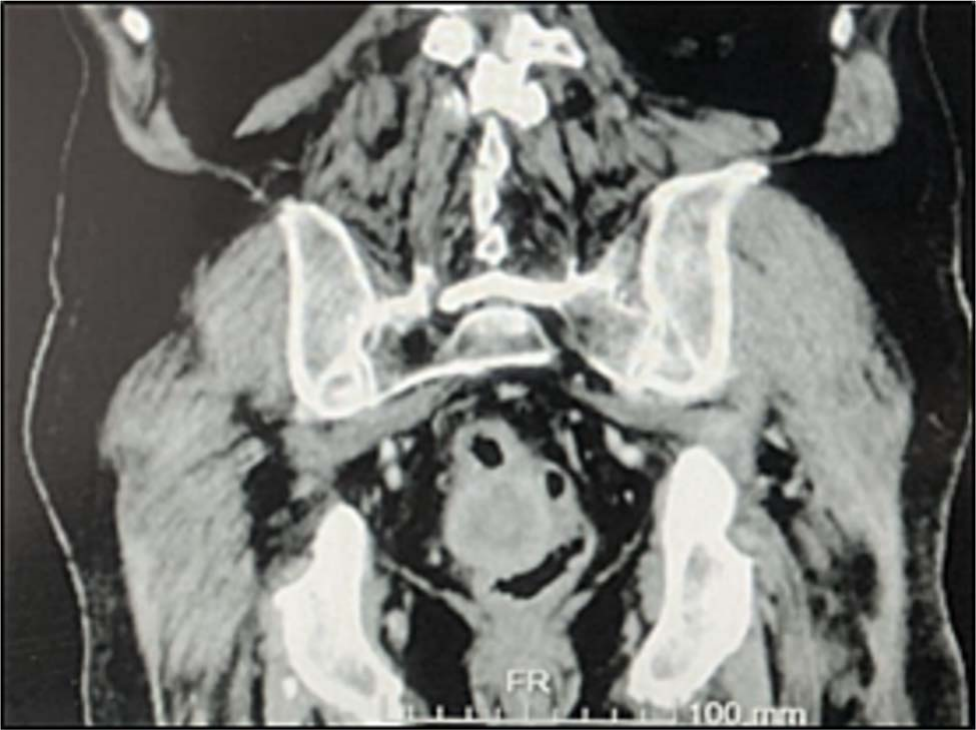
Rectal lesion (coronal plane).

A contrast-enhanced chest-abdominal-pelvic computed tomography (CT) scan revealed a solid, multilobular mass, with approximately 8 × 6 × 8 cm, likely originating from the SB, with asymmetric wall thickening, heterogeneous contrast enhancement, areas consistent with necrosis and central calcification (Figs 1 and 2), suggestive of GIST. Simultaneously, a solid nodular lesion was identified on the anterior wall of the rectum, exhibiting exophytic growth with a heterogeneous texture, well-defined borders and measuring 5 × 3 cm, without adjacent fat infiltration, raising suspicion of another GIST (Fig. 3).

The patient experienced several episodes of GI bleeding and was admitted for investigation.

**Figure 4:**
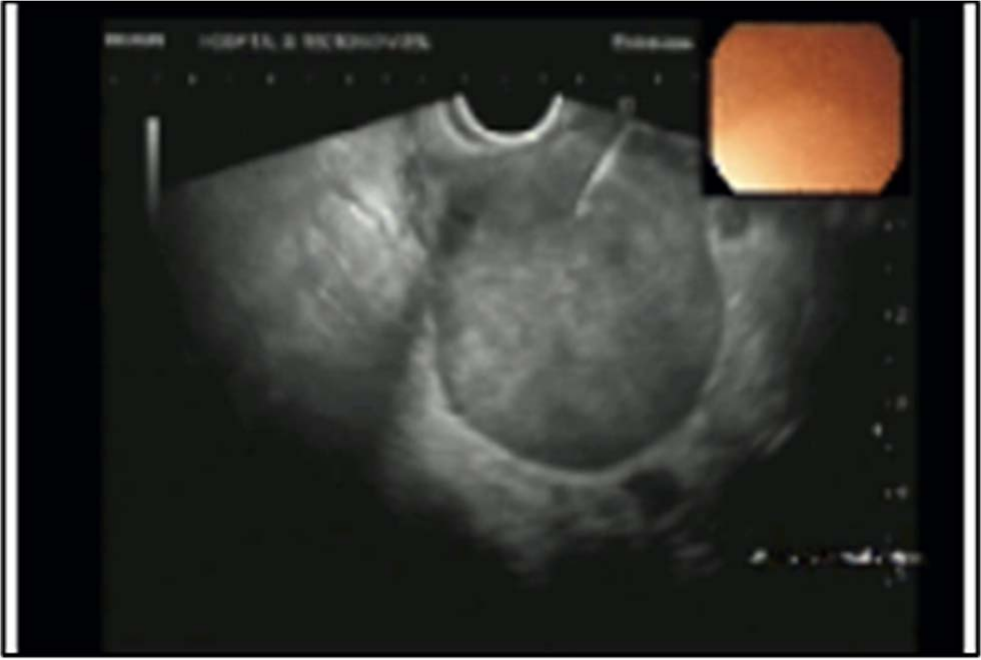
Endorectal ultrasound.

Rectal endoscopic ultrasound revealed an extrinsic lesion on the rectal wall, within the mesorectum (Fig. 4). The biopsy was suggestive of adenocarcinoma.

**Figure 5:**
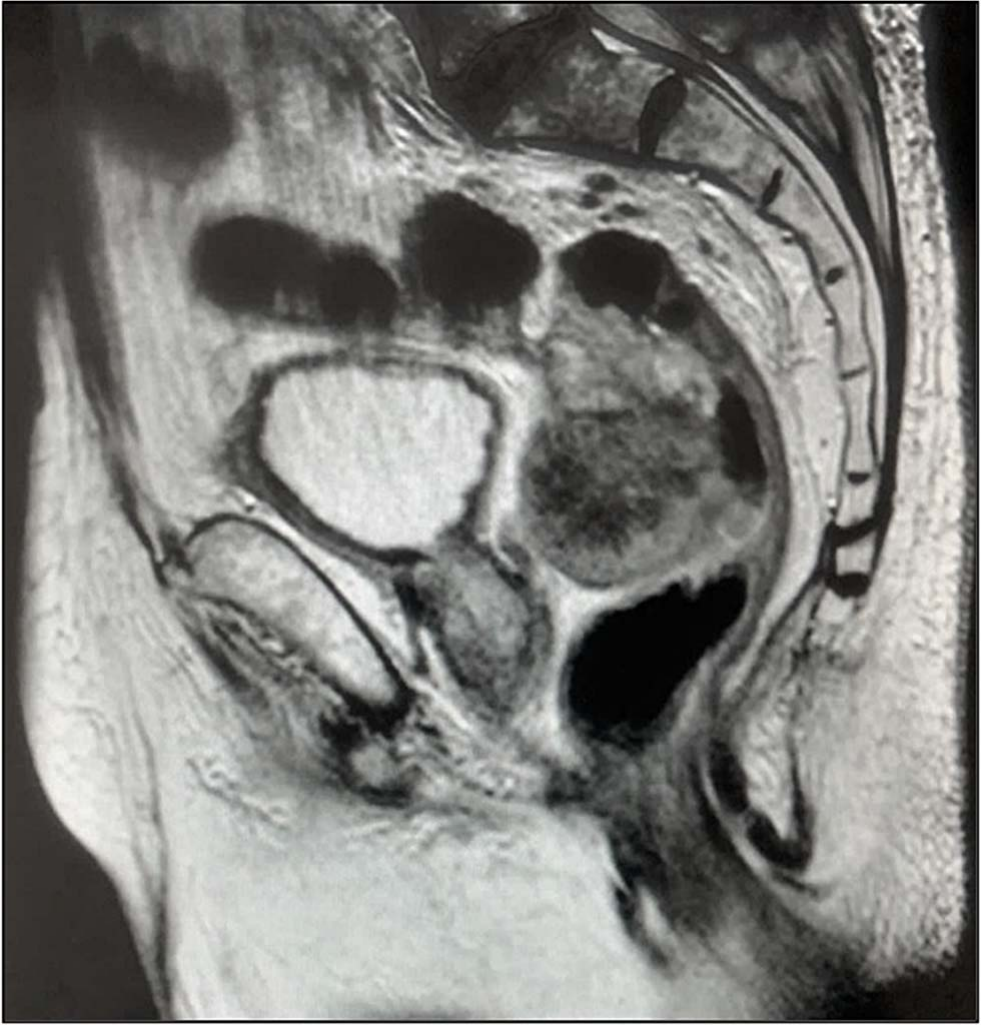
Mesorectal lesion (T2—Sagital plane).

**Figure 6:**
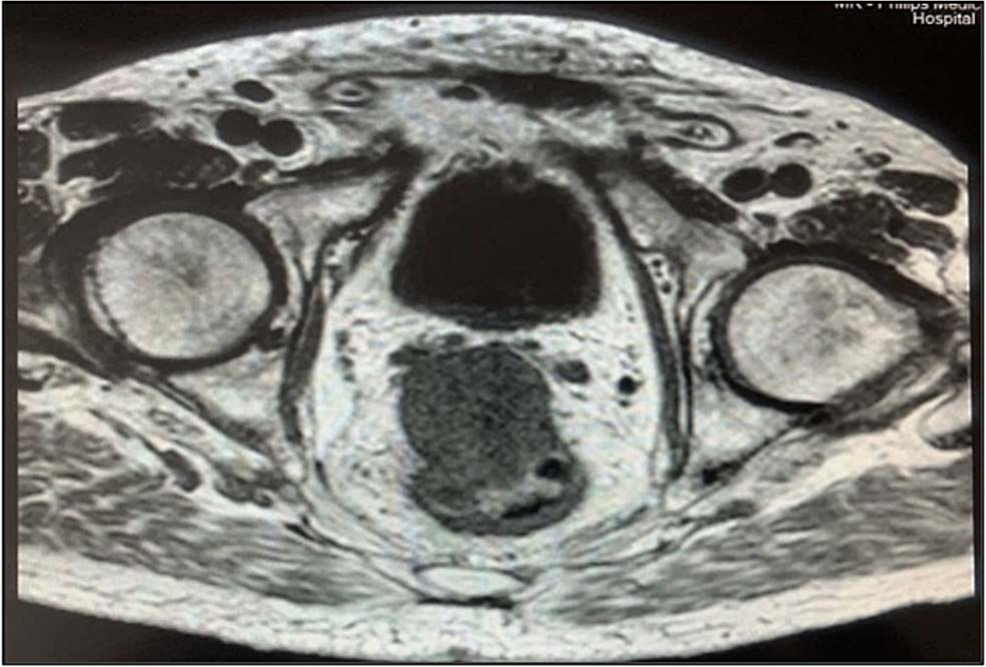
Mesorectal lesion (T1—Axial plane).

Pelvic magnetic resonance imaging (MRI) revealed a extrarectal lesion located 8 cm from the anal verge, with 4 × 4 cm, suspicious of tumoral deposit in the mesorectum (Figs 5 and 6).

CEA and CA 19–9 levels were normal.

Therefore, the case was likely a malignant SB tumor with a single mesorectal metastasis. In multidisciplinary meeting the decision was to proceed with surgical intervention, given the disease’s resectability and recurrent GI bleeding requiring multiple transfusions (a total of eight units of packed red blood cells).

**Figure 7:**
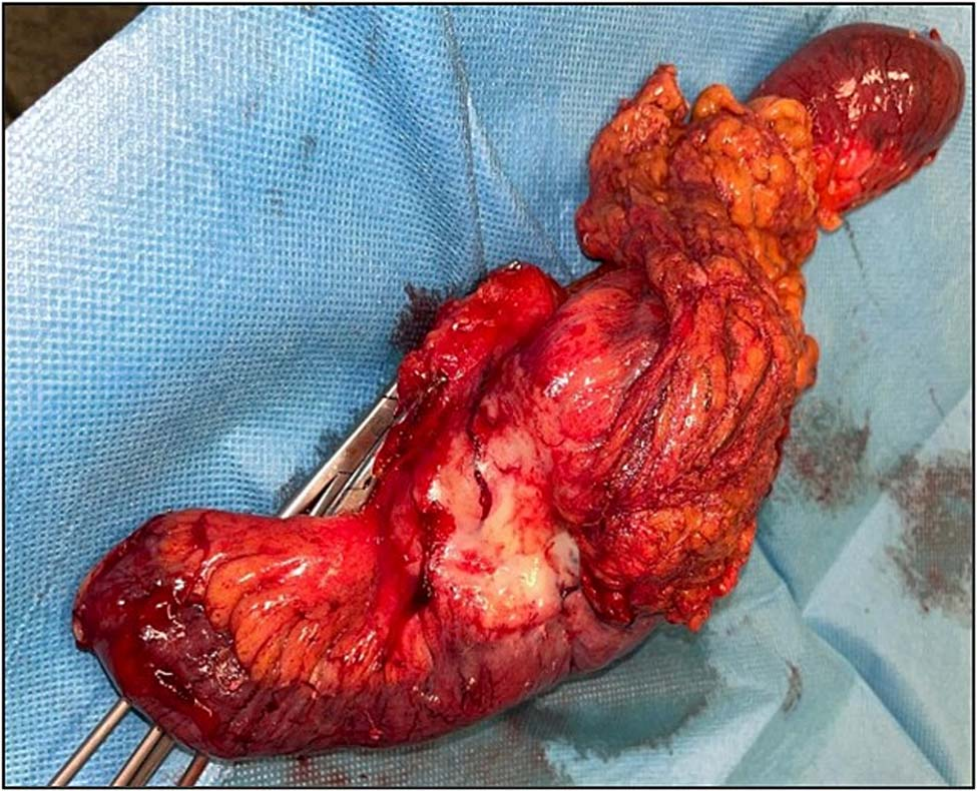
Jejunal adenocarcinoma—specimen.

The patient underwent an exploratory laparotomy with segmental enterectomy of approximately 30 cm of jejunum (Fig. 7) and Hartmann’s procedure with partial mesorectal excision. The postoperative course was complicated with a pelvic abscess, managed with CT guided percutaneous drainage and antibiotic therapy. The patient was discharged on the 23rd postoperative day.

The histopathological examination revealed:

(1) jejunal mucinous adenocarcinoma with serosal involvement, lymphovascular and perineural invasion, without metastasis in the 16 lymph nodes examined; and

(2) mesorectal tumoral deposit, without ulceration of the rectal mucosa, with lymphovascular and perineural invasion and no metastasis in the 19 lymph nodes examined, consistent with metastasis from SB adenocarcinoma.

Clear margins were observed in both specimens. Staging: pT4 N0 LVI+ PNI+ R0 M1.

In multidisciplinary meeting it was decided to initiate adjuvant chemotherapy. Following the second cycle of systemic therapy, the patient presented to the ED with rectal bleeding. Rectoscopy revealed a hemorrhagic lesion at the apex of the rectal stump. Endoscopic therapy with argon plasma was performed.

**Figure 8:**
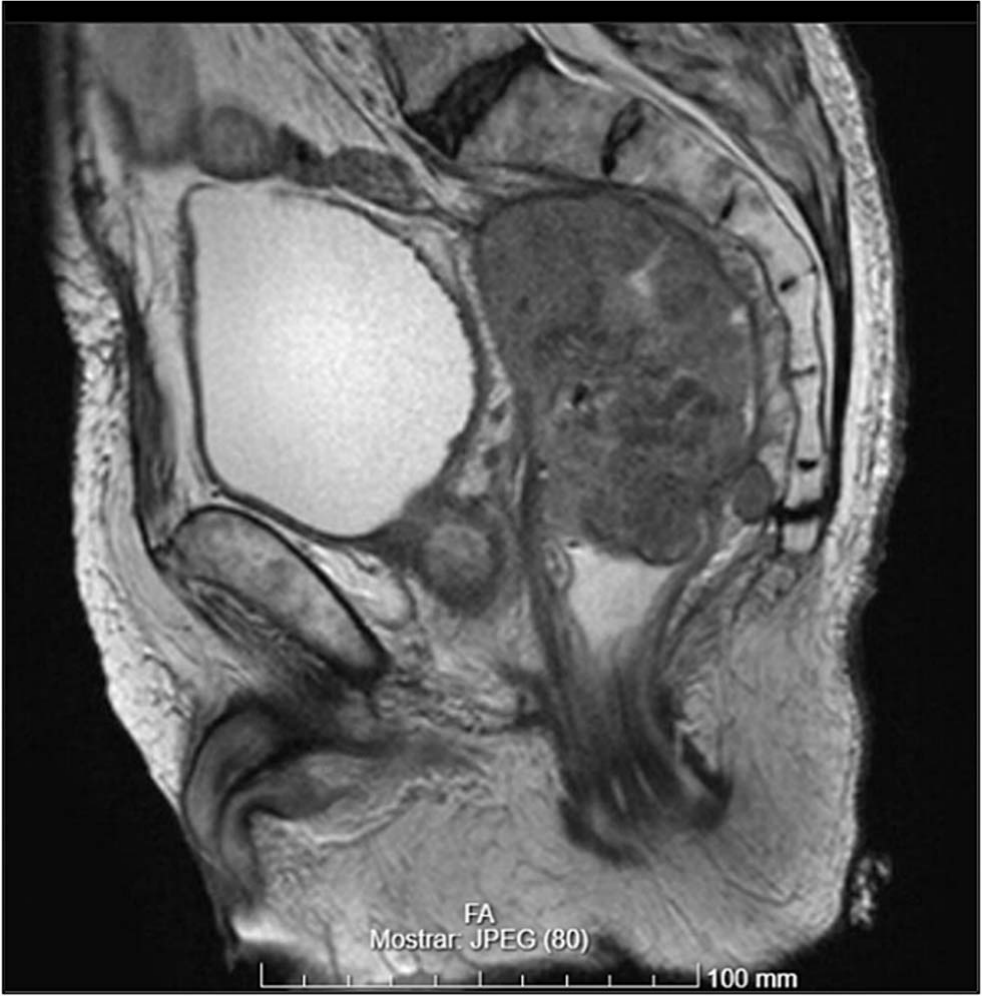
Pelvic recurrence (MRI—T2 sagittal plane).

**Figure 9:**
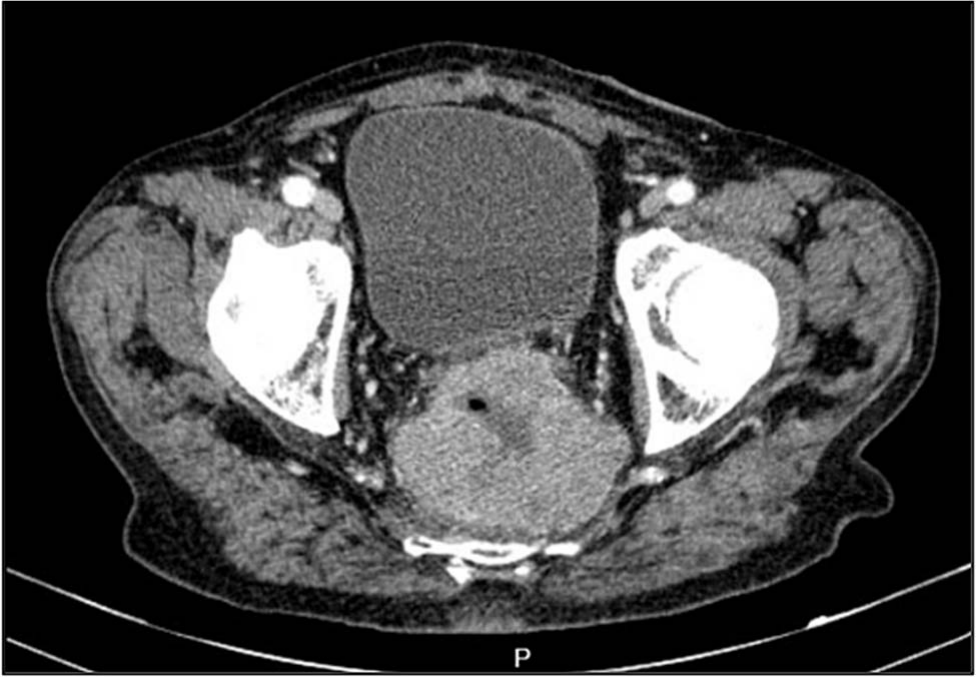
Pelvic recurrence (CT scan—Axial plane).

**Figure 10:**
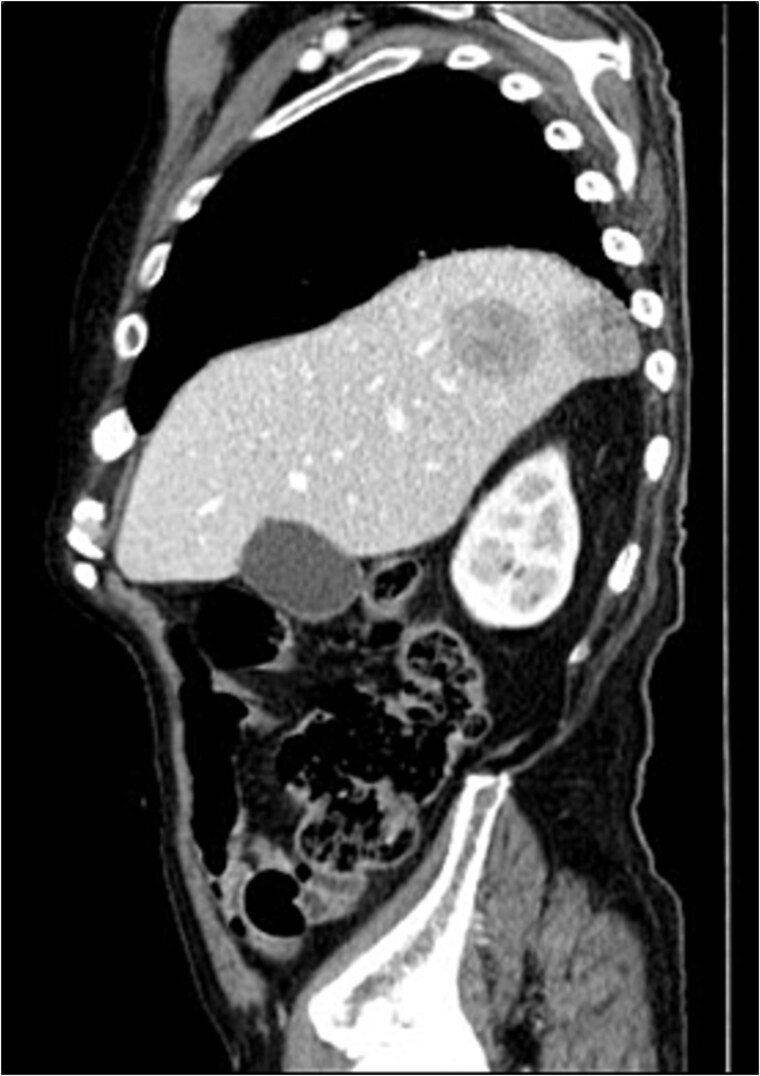
Liver metastasis (CT scan—Sagittal plane).

CT scan and pelvic MRI revealed:

- A mass measuring 9 × 8 cm near the rectal stump, consistent with tumor recurrence (Figs 8 and 9);

- Two liver metastases located in segments seven and eight, each measuring 5 cm (Fig. 10).

Given the gradual decline in the patient’s overall condition, palliative treatment became the primary focus of care and the patient passed away 7 months later.

## Discussion

SB neoplasms are rare, but most of them are malignant. SB adenocarcinomas are more frequent in the duodenum (52% to 57%), followed by jejunum (18% to 29%) [9]. These tumors are more common in men and between the ages of 55 and 65 [12, 13].

Adenocarcinomas generally arise from premalignant adenomas. Excessive consumption of alcohol, tobacco, processed, or red meats, and the presence of obesity are recognized risk factors [4, 14].

They usually present with symptoms of obstruction and anemia [10]. Gastrointestinal bleeding occurs in 23%–44% of cases [2, 15]. In cases of OGB evaluation of the SB should be conducted [16–18]. Approximately 35%–40% of patients with SB adenocarcinoma are reported to have distant metastases at the time of diagnosis [19].

The treatment approach of these tumors depends on initial staging [9].

In localized disease, segmental enterectomy and resection of the corresponding mesentery, with free margins and analysis of at least 8 to 10 regional lymph nodes, is the standard surgical treatment [10, 20]. Nevertheless, disease recurrence is common. Therefore, adjuvant chemotherapy is proposed to reduce recurrence, mainly if lymph node involvement is present [7, 21]. Currently, the NCCN Clinical Practice Guidelines in Oncology recommends systemic therapy for advanced SB adenocarcinoma. The 5-year overall survival (OS) rates are of 50%–60% for stage I, 39%–55% for stage II and 10%–40% for stage III [8].

Regarding metastatic disease, the prognosis is poor, with reported OS rate at 2 years of 5%–10%, at 5 years of 3%–5% [19, 22]. In these cases, systemic therapy is usually the first line treatment option. In well-selected patients, metastasis resection appears to be associated with improved OS. This improvement was seen across several metastatic sites, including liver, lung, and peritoneum [11].

In our case, initial surgical treatment was indicated due to refractory anemia and oligometastatic disease, related to a single resectable nodule in the mesorectum.

## Conclusion

Small bowel adenocarcinoma is rare, aggressive, difficult to diagnose and treat. Early detection may improve survival despite poor prognosis. The authors report a case of a jejunal adenocarcinoma with a single mesorectal metastasis, a previously unreported site for metastasis of this tumor type. Although our patient died within one year of diagnosis, it is important to outline the demand of its workup and treatment decisions.
